# Macular hole secondary to Valsalva retinopathy after doing push-up exercise

**DOI:** 10.1186/1471-2415-14-98

**Published:** 2014-08-12

**Authors:** Zheng-gao Xie, Su-qin Yu, Xi Chen, Jun Zhu, Fang Chen

**Affiliations:** 1 Department of Ophthalmology, Clinical Medical College of Yangzhou University, No.98, Nantong West Road, Yangzhou, Jiangsu Province 225001, China; 2 Department of Ophthalmology, Subei People’s Hospital of Jiangsu Province, No.98, Nantong West Road, Yangzhou, Jiangsu Province 225001, China; 3 Department of Ophthalmology, the Affiated First People’s Hospital of Shanghai Jiao Tong University, No.100, Haining Road, Shanghai 200080, China

**Keywords:** Optical coherence tomography, Valsalva retinopathy, Macular hole

## Abstract

**Background:**

Valsalva retinopathy and traumatic macular hole are common conditions, but macular hole secondary to Valsalva retinopathy is rarely reported.

**Case presentation:**

A 34-year-old healthy man suffered Valsalva retinopathy after doing push-up exercise. During his follow-up visits, the best-corrected visual acuity (BCVA) measurements, fundus examinations and spectral-domain optical coherence tomography (SD-OCT) tests were performed. Three months later, the premacular hemorrhage was noticeably absorbed with an improvement of visual acuity. SD-OCT showed a lamellar macular hole with intact but thickened internal limiting membrane (ILM) with vitreal tractions on surface of the macular. Nine months after the first visit, his vision acuity was 20/25. The fundus examination showed a complete absorption of the macular hemorrhage. SD-OCT showed that the lamellar macular hole has enlarged, with thickened ILM on the surface. Seventeen months after the onset, the BCVA, fundus examination results and OCT findings were stable.

**Conclusions:**

Macular hole secondary to Valsalva retinopathy had been rarely reported and its mechanism needs further understanding. SD-OCT can be used to observe the evolvement of Valsalva retinopathy.

## Background

Valsalva retinopathy was first described in 1972 by Duane as a pre-retinal hemorrhagic retinopathy secondary to a sudden increase in intrathoracic pressure. Following a Valsalva maneuver, a sudden rise in intraocular venous pressure causes retinal capillaries to rupture [1]. The prognosis is generally good, with spontaneous resolving of the hemorrhage in most cases, though it may take several months [2]. Observation, vitrectomy or neodymium:YAG (Nd:YAG) laser membranotomy are the current treatment options [3,4]. We reported a case of Valsalva retinopathy combined with an incidental lamellar macular hole.

## Case presentation

**Figure 1:**
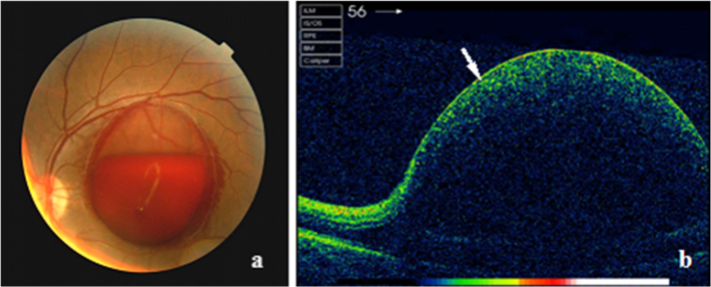
**Fundus photography and SD-OCT scan of the patient.** Fundus photograph showed a massive well-circumscribed premacular hemorrhage of 3-discdiameter underneath a transparent membrane **(a)**. There was a fluid level within the hematoma. Corresponding SD-OCT revealed a dome-shaped elevated lesion with a hyperreflective surface (white arrow) and a hypo-reflective area beneath **(b)**.

**Figure 2:**
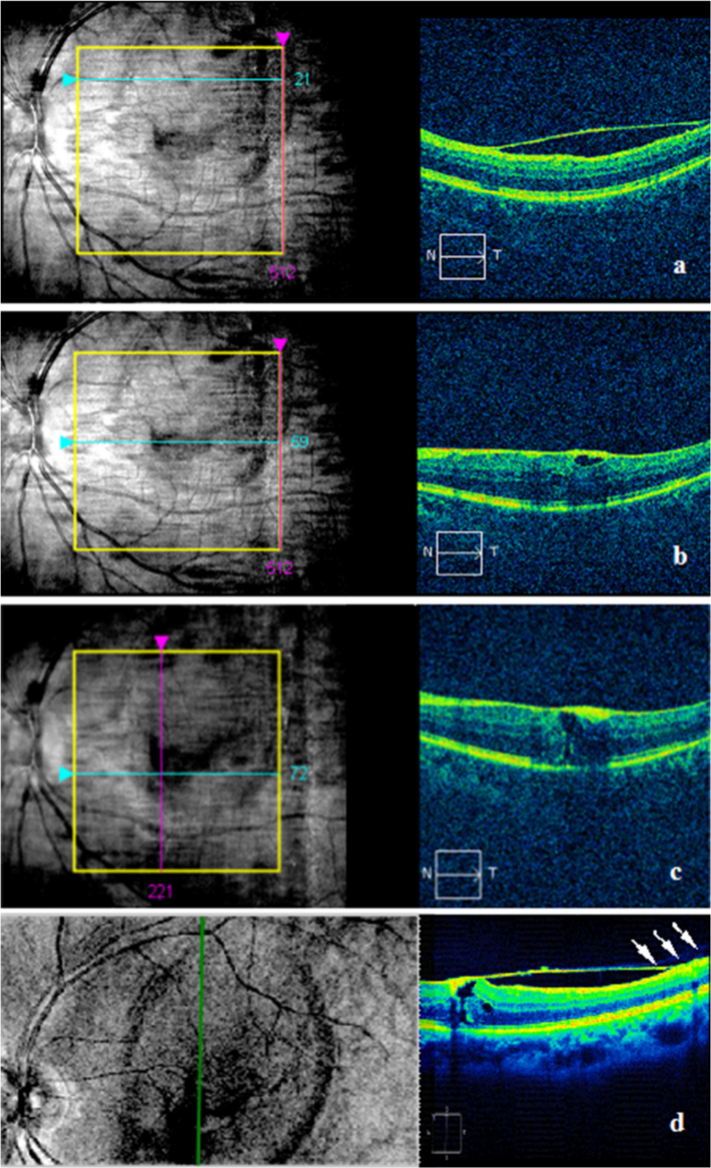
**SD-OCT scan of the patient at three months.** SD-OCT scan showed the ILM on the top of macular was still detached **(a)**. SD-OCT examination also revealed thickened hyper-reflective ILM in macular fovea, the thickened nerve fiber layer (NFL) with cysts formed under it **(b)**, a tangential traction on the fovea by the elevated ILM and a lamellar macular hole of 117 um formed **(c, d)**. Vertical OCT scan demonstrated a tangential traction on the fovea by detached and thickened ILM, and the posterior hyaloid membrane, characterized by hypo-reflectivity (white arrow), was detected above the ILM **(d)**.

**Figure 3:**
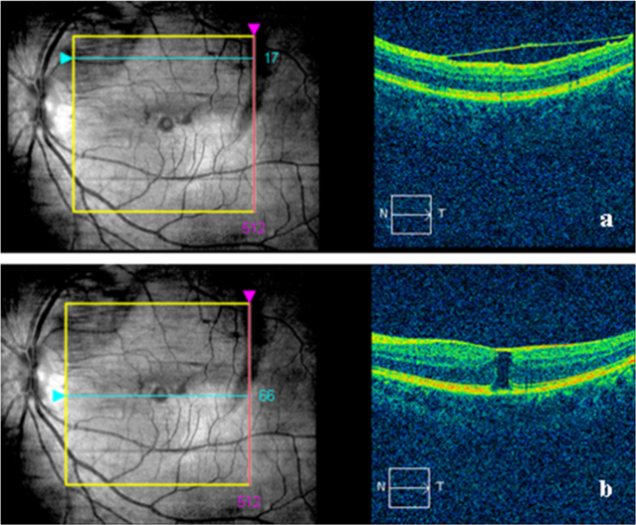
**SD-OCT scan of the patient at nine months.** Nine months after the initial visit, SD-OCT scan showed detached ILM on the top of macular **(a)**, and the enlarged lamellar macular hole of 296 um with thickened ILM on the top **(b)**.

**Figure 4:**
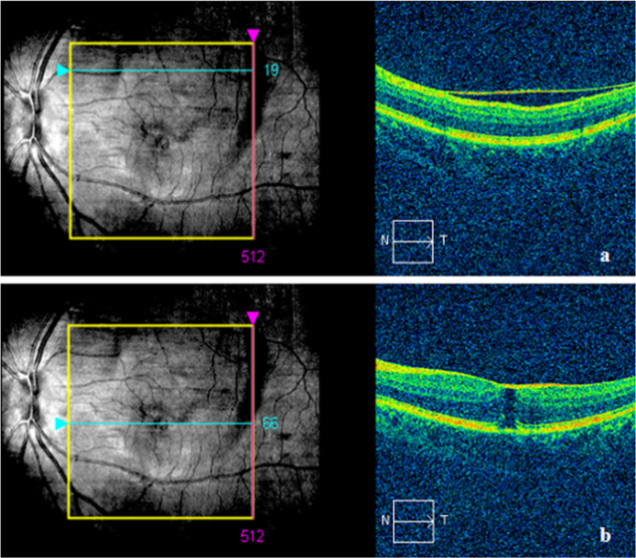
**SD-OCT scan of the patient at seventeen months.** SD-OCT scan showed the detached ILM **(a)** and the lamellar macular hole (288 um) **(b)** were akin to 9 months.

A 34-year-old healthy man suffered a sudden visual loss in his left eye after doing push-up exercise. His medical history was unremarkable. The best-corrected visual acuity (BCVA) of the left eye on presentation was counting fingers and 20/20 right. The findings of ocular motility, external and anterior segment examinations were unremarkable in both eyes. Intraocular pressures were normal in both eyes. The fundus examination showed a massive well-circumscribed pre-macular hemorrhage with a fluid level underneath a transparent membrane in the left eye (Figure 1a). The spectral-domain optic coherence tomography (SD-OCT) (Carl Zeiss, Cirrus HD-OCT, Germany) revealed a dome-shaped elevated lesion with a hyper-reflective surface and a hypo-reflective area beneath (Figure 1b).Management options including observation, laser membranotomy, and vitrectomy were discussed with the patient. The patient preferred observation. Three months after the initial visit, his visual acuity of the left eye was improved to 20/160. Dilated funduscopic examination showed remarkable absorption of the hemorrhage, leaving a dome-shaped preretinal membrane. SD-OCT revealed that the internal limiting membrane (ILM) was thickened and hyper-reflective, and the nerve fiber layer (NFL) had intraretinal cystic changes at the fovea (Figure 2a, b). OCT scan also revealed a tangential traction on the fovea by the elevated ILM and a lamellar macular hole (Figure 2c, d). The posterior hyaloid membrane was detected above the ILM without complete posterior vitreous detachment (Figure 2d). Nine months after the initial visit, his visual acuity was further improved to 20/25 with complete resolution of the macular hemorrhage. SD-OCT showed the enlarged lamellar macular hole with thickened ILM on the surface (Figure 3a, b). Since the patient experienced no metamorphopsia and was satisfied with his visual acuity, no interventional treatments were performed. On his most recent visit, seventeen months after the onset, BCVA remained at the level of 20/25 in the affected eye. The fundus examination and OCT findings were stable (Figure 4a, b).

## Conclusions

Valsalva retinopathy may occur during various activities including severe coughing, forced nose blowing, suffocating, crying, weightlifting, tug-of-war playing, vomiting, military training, hard blowing through mouth, hard straining, thoracic abdominal extrusion, pregnancy, forced childbirth, sexual activity, and colonoscopy, etc [5]. In this case, the patient had a sudden vision lose after doing push-up exercise. Fundus and OCT examinations revealed sub-ILM premacular discoid fresh hemorrhage. We speculate that while doing push-up exercise, increased intra-thoracic and/or intra-abdominal pressure(s), which induced a rupture of the capillaries around the macular leading to the sub-ILM hemorrhage.

The exact anatomic location of the premacular hemorrhage in Valsalva retinopathy, whether it is at sub-ILM or subhyaloid or a combination of the both, has been disputed in the literature [6]. We believe that the exact location of the premacular hemorrhage cannot be accurately determined by ophthalmoscopy. Kwok *et al*. [7] provided histologic evidence of a sub-ILM location in a Valsalva hematoma. With the advent of OCT, it has been found that sub-ILM hemorrhage is more common than subhyaloid hemorrhage [8,9]. Only if both the ILM and the posterior hyaloid membrane are visible on OCT, can the location of hematoma be ascertained. We believe that this peculiar case is an example of sub-ILM hemorrhage, since OCT demonstrated the ILM and the posterior hyaloid membrane as two distinct layers. Nine months later, the hemorrhage was completely resolved with a dramatic improvement of patient’s visual acuity. However, OCT scan revealed a discontinuity of neurosensory retina except ILM in the fovea. There are two possible explanations for his good visual acuity outcome. First, it is an outer retinal lamellar macular hole rather than a full-thickness macular hole. Patients may preserve good vision in the case of a lamellar hole. Secondly, the patient may have developed a “VERY NEAR FOVEA” paracentral eccentric fixation, which is very close to the center of the fovea. This paracentral fixation should be confirmed with microperimetry, however, we did not have this technology available during our data collection.

To the best of our knowledge, there is no report of Valsalva retinopathy combined with a lamellar macular hole. Kwak *et al*. [10] reported a macular hole followed vitrectomy for Valsalva retinopathy. Ulbig *et al*. [11] reported a macular hole identified after laser treatment. In this case, the patient did not receive any interventional treatment. The lamellar macular hole was developed spontaneously. Although the mechanism of macular hole formation in Valsalva retinopathy is not fully understood, there are several possibilities. First, the lamellar macular hole could be induced directly by a sizable amount of hemorrhage breaking through the neurosensory layers. Secondly, the thickened ILM observed after resolution of the premacular hemorrhage may have created a tangential traction on the fovea. Thirdly, the massive and long-term hemorrhage put the ILM under high tension, which may make ILM hard to reattach to the retina [12]. The partial detached ILM on the surface of the retina may produce traction on the macula as well. Finally, SD-OCT revealed the surface of the NFL under the detached ILM had an abnormal rough appearance. Besides the acute traumatic detachment between the two layers (ILM-NFL), the toxicity of the long-lasting blood may have contributed to such an abnormality [13].

Therapeutic options in Valsalva retinopathy include conservative management, vitrectomy, and laser membranotomy. Hemorrhage could be self-absorbed in most cases and the conservative treatment is commonly used. However, spontaneous absorption of the preretinal blood is very slow. Epiretinal membrane formation and toxic effect of dissolving hemoglobin has been suggested after long-standing contact between blood and retina [14,15]. Therefore, laser puncturing by Nd:YAG laser is recommended to drain the entrapped blood through a focal opening into the vitreous cavity where it is absorbed more rapidly [3,11]. Nd:YAG laser treatment for Valsalva retinopathy is generally effective and safe. However, complications may occur occasionally. The long-term complications of Nd:YAG laser membranotomy include macular hole, retinal detachment, epiretinal membrane formation, and persistent unsealed internal limiting membrane [3,7,12]. The patient in this case was afraid of the risks of laser treatment and vitreous surgery, so conservative treatment was taken, and it took a longer absorption time. If YAG laser had been performed, the sub-ILM hemorrhage would have been absorbed more rapidly; the tension of the ILM might have been relieved earlier, and the formation of the macular hole might have been prevented.

In conclusion, SD-OCT can be used to observe the development of Valsalva retinopathy, and suitable treatments should be adopted timely to prevent complications.

## Consent

Written informed consent was obtained from the patient for publication of this case report and any accompanying images. A copy of the written consent is available for review by the editor-in-chief of this journal.

## Abbreviations

SD-OCT: Spectral-domain optic coherence tomography; ILM: Internal limiting membrane; NFL: Nerve fiber layer; BCVA: Best-corrected visual acuity; NFL: Nerve fiber layer.

## Competing interests

The authors declare that they have no competing interests.

## Authors’ contributions

ZGX and SQY contributed to the clinical management of the patient, collecting clinical information of the patient, literature search, planning the case report and writing the article. XC and JZ performed retinal examinations and OCT evaluation, etc. FC was the main physician responsible for the patient and suggested to report this case and participated in writing the manuscript. All authors has read and approved the final manuscript.

## Pre-publication history

The pre-publication history for this paper can be accessed here:

http://www.biomedcentral.com/1471-2415/14/98/prepub
